# Preparation and performance of semiconductor device bonding joints based on Cu@Sn@Ag preform

**DOI:** 10.1039/d3ra06909e

**Published:** 2023-12-07

**Authors:** Honghui Zhang, Hongyan Xu, Tianwen Wang, Sheng Wang

**Affiliations:** a Xinyang Vocational and Technical College Xinyang 464000 China zhh20080115@163.com; b Micro-nano Fabrication Technology Department, Institute of Electrical Engineering, Chinese Academy of Sciences Beijing 100190 China hyxu@mail.iee.ac.cn

## Abstract

Herein, a 110 A commercial, Si continuous current diode with high heat dissipation power is attached to Cu@Sn@Ag preform, formed by electroplating and physical vapor deposition, and pressed into a preformed sheet under a pressure of 5–10 MPa. The prepared dense three-dimensional network of Cu/Cu_3_Sn/Ag_3_Sn joint, based on Cu@Sn@Ag preform obtained using transient liquid-phase diffusion soldering technology, can withstand high temperatures up to 600 °C in power device applications. The mechanical and thermal performance and power cycle reliability of the Cu@Sn@Ag joint are investigated and comparatively analyzed with PbSn5Ag2.5 joints. The results show that the average shear strength of the Cu@Sn@Ag joint is ∼35 MPa, which exceeds that of PbSn5Ag2.5 solder joint, and is similar to that of sintered nano-silver solder joint; the minimum thermal resistance of the corresponding device is ∼0.18 K W^−1^, near to that of PbSn5Ag2.5 joint. The growth rate of the forward voltage drops below 2% following 150 000 active power cycles, with a junction temperature difference below 60 °C, meeting the requirements of reliability test standards for vehicle specifications. It is concluded that the performance and power cycle reliability of the Cu@Sn@Ag joint are better than those of the PbSn5Ag2.5 joint.

## Abstract 2

Herein, a 110 A commercial, Si continuous current diode with high heat dissipation power is attached to Cu@Sn@Ag preform, formed by electroplating and physical vapor deposition, and pressed into a preformed sheet under a pressure of 5–10 MPa.

## Graphical abstract

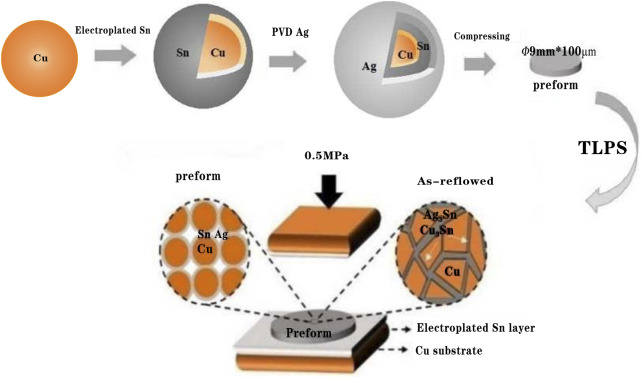

## Introduction

A power module, an important energy conversion and control device, is extensively employed in various fields including aerospace, advanced energy, electric vehicles, 5G communication, and photovoltaic power generation. Owing to the rapid development of power modules toward high integration and high-power density, their application temperatures rise to 600 °C. However, the high-temperature-resistant soldering materials employed for power devices have difficulty in exerting the original excellent performance of power devices, particularly for SiC or GaN chips, which pose a formidable challenge for chip soldering materials. Therefore, the development of high-temperature-resistant and high-reliability lead-free soldering materials is imperative for settling this issue.^1–4^

The traditional high-lead-content alloys, including Au–Sn and Sn–Sb–Ag, can easily cause chip warpage and deformation under high chip junction temperatures, and they are unable to meet the requirements of high service temperatures for power devices. Although sintered nanosilver shows comprehensive high performance, the high pressure during the soldering process and the occurrence of silver migration during service easily induce high porosity in the solder layer and deteriorate the joint properties. Additionally, the high cost precludes its popular application. Transient liquid-phase diffusion soldering (TLPS) combines the characteristics of diffusion welding and brazing. Therefore, it is possible to realize low-temperature bonding and high-temperature service and is a viable replacement for high-lead-content alloy solder. Further, it has tremendous prospects in high-temperature packaging for the new-generation power devices represented by SiC.^5^ Chu *et al.*^6^ have employed sandwiched Cu/Sn/Cu and Ni/Sn/Ni material structures and low-temperature TLPS (LT TLPS) technology to prepare pure interfacial intermetallic compound (IMC) joints. These joints are soldered by sandwich-structured metal layers comprising a low-melting-point interlayer, such as Sn or In, and a surface layer of high-melting-point metals, such as Cu, Au, or Zn.^7–9^ High-temperature-resistant IMCs are formed as the interconnecting joint *via* liquid–solid interdiffusion, though the long diffusion channel of the Cu/Sn/Cu structure extends the interfacial reaction time and makes it difficult to extend the interface layer thickness. Further, pure IMC joints increase the elastic modulus and reduce the fracture toughness, which are not conducive to reliable running at high temperatures.^10^ For these challenges, composite-powder-solder-paste-based TLPB technology for the preparation of IMC high-temperature joints has been extensively studied.^11–13^ This technology is advantageous as the Cu/Sn particles are uniformly mixed and fully intercontacted, and the molten Sn completely infiltrates the Cu particles, which significantly shortens the diffusion channel and strengthens the reaction kinetics. Subsequently, the isothermal diffusion alloying reaction generates homogeneous Cu_6_Sn_5_/Cu_3_Sn compounds. H. Grave *et al.*^14^ successfully prepared complete Cu_6_Sn_5_ and Cu_3_Sn joints with a joint thickness below 10 μm by employing this technology, and the thermoelectric properties of the joint are comparable to those of pure silver. However, one of the challenges with this technology is that the structure of the above-prepared high-temperature-resistant joints persists as the pure IMC phase, with high elastic modulus, poor stress absorption capacity, and susceptibility to brittle fracture at the joint interface. Further, challenges that reduce the reliability have not been overcome yet.^15–17^ Therefore, the joint structure needs to be improved to strengthen the toughness of the joint.

To address these challenges, a dense core–shell structure of Cu@Sn@Ag preform has been developed *via* electroplating and physical vapor deposition using low-cost Cu particles with a high thermal and electrical conductivity. By refining the micro- and nanometer Cu particle size and the thickness of the Sn/Ag coating layer, the structure, strength, and toughness of the soldering joint can be controlled. This provides an important theoretical basis for the preparation of high-temperature-resistant joint technology *via* TLPS of the Cu/Sn/Ag system. The thermal, mechanical, and electrical performance of the assembled Si device has been tested and compared with those of the commercial PbSn5Ag2.5 preform to verify the reliability of the Si device assembled with the Cu@Sn@Ag preform.

## Materials and methods

### Preparation of Cu@Sn@Ag preform

Three kinds of Cu powder, procured from Beijing Institute of Nonferrous Metals, with different particle sizes of <15, 15–25, and 25–50 μm, were selected and mixed according to the mass ratio of 1 : 3 : 2 and deoxidized with ethanol hydrochloric acid solution. Further, the activated Cu powder was uniformly and densely plated with a 2–3 μm-thick Sn layer in a prepared solution containing stannous methyl sulfonic acid, methylsulfonic acid, resorcinol, and emulsifier. Subsequently, the Cu@Sn core–shell powder was prepared after ultrasonic cleaning with deionized water and ethanol 3–5 times before being dried in an oven. Next, the prepared Cu@Sn core–shell powder was coated with a 1–2 μm-thick Ag layer outside the Sn layer through physical vapor deposition (PVD) method to obtain the Cu@Sn@Ag composite powder. Finally, the Cu@Sn@Ag particles were compressed under a pressure of 10 MPa for 5 min to obtain the preform with dimensions of *φ* 9 mm × 100 μm.

### Preparation of Cu@Sn@Ag TLPS high-temperature-resistant joint

**Fig. 1:**
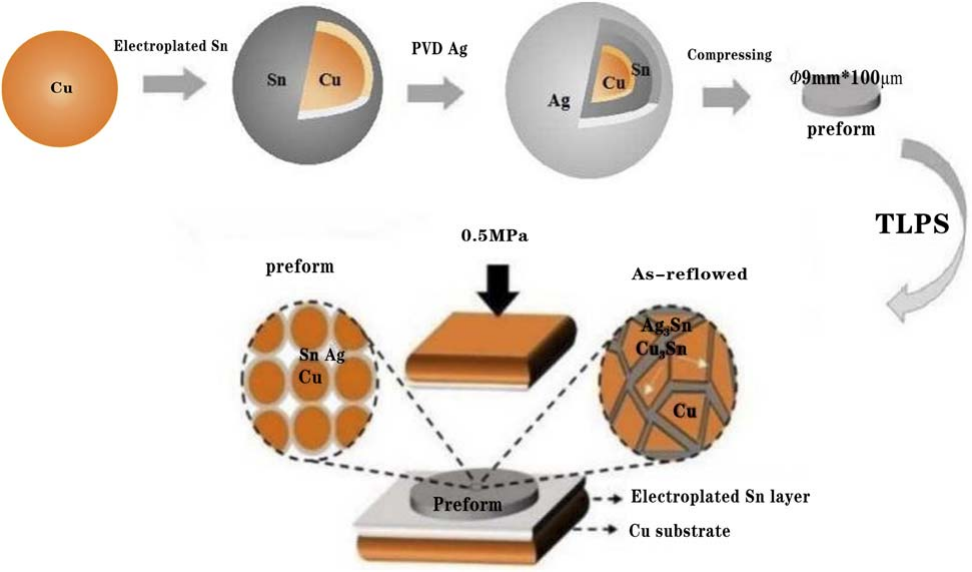
Schematic configuration of the preform and joint preparation.

To improve the consistency of the interconnection interface between the preform and the Si chip, an additional layer of Sn (1–2 μm) was plated on the surface of the preform. Further, the flux was dripped on the surface of the Direct Bond Copper (DBC) board to prevent the substrate oxidization during the welding process. The Si chip, preform, and DBC board were fixed with a fixture and placed in a welding furnace. The joint, comprising a dense three-dimensional network of Cu/Cu_3_Sn/Ag_3_Sn, was obtained after heating at 280 °C for 20 min. The schematic configuration of the preform and joint preparation is shown in Fig. 1.

**Fig. 2:**
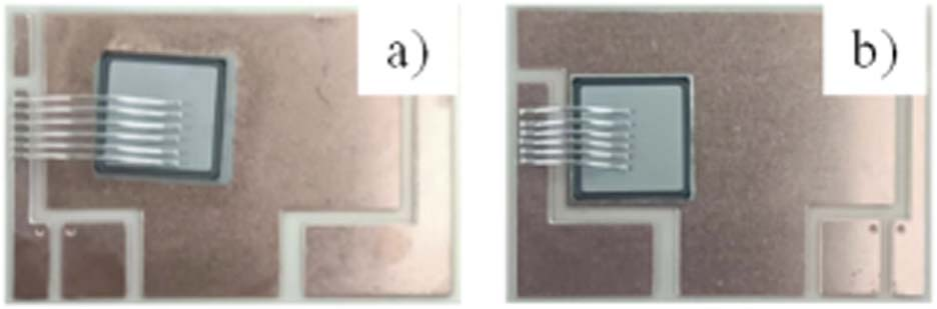
Photographs of Si chips assembled using different solders. (a) Cu@Sn@Ag preform welding. (b) Commercial PbSn5Ag2.5 preform welding.

Following the soldering process, the flux residue on the surface of the Si chip was ultrasonically cleaned in ethanol, and 6–8 aluminum wires were welded using ultrasonic assistance on the surface of the chip to complete the assembly of the Si device. Photographs of the assembled Si chips are displayed in Fig. 2. The entire Si device consists of six layers, namely, the layers of lower copper-clad DBC, DBC ceramic, upper copper-clad DBC, preform, and chip, besides the Al bonding line from bottom to top.

## Results and discussion

### Composition and morphology of Cu@Sn@Ag core–shell structure powder

**Fig. 3:**
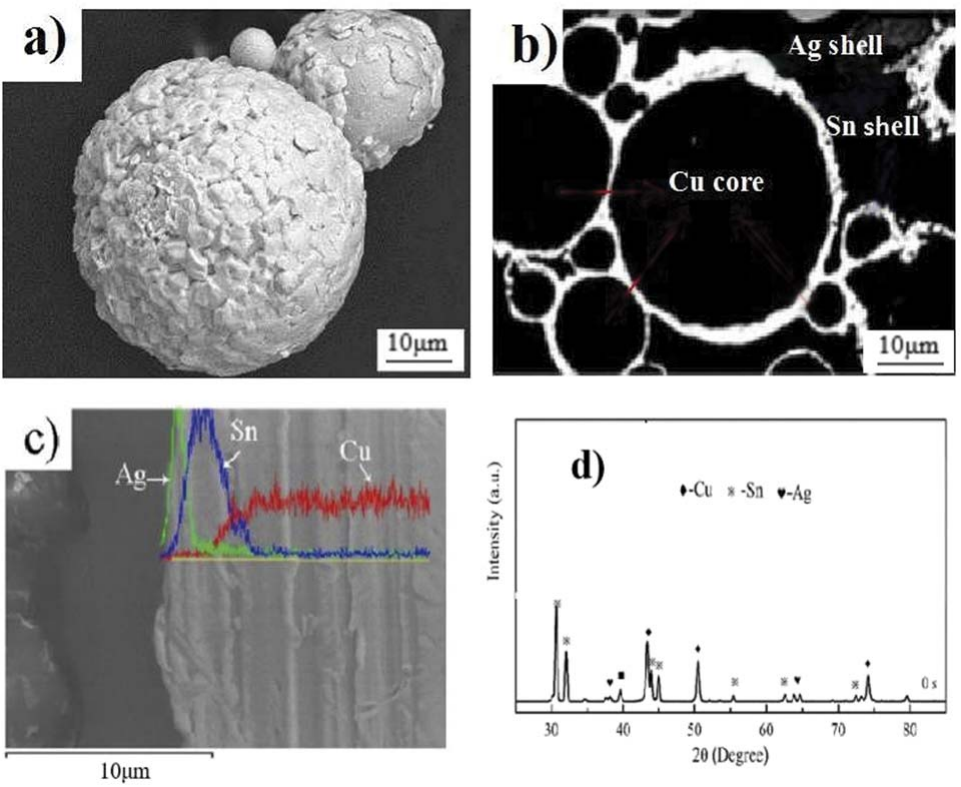
(a) Surface morphology, (b) profile morphology, (c) EDX results, and (d) XRD for of Cu@Sn@Ag core–shell structure powder.

The surface morphology and cross-sectional morphology of the Cu@Sn@Ag powder prepared *via* PVD after electroplating are depicted in Fig. 3a and b. The Cu@Sn@Ag powder is silver–white, and the Sn shell is covered uniformly on the Cu core with a thickness of ∼2–3 μm, whereas the outer Ag shell with a thickness of ∼1–2 μm is uniformly coated on the surface of the Cu@Sn powder. The core–shell structure of the Cu@Sn@Ag powder has been proved using energy dispersive X-ray spectroscopy (EDX) line-scanning analysis at the Ag–Sn–Cu interface; the elements Cu, Sn, and Ag were detected *via* EDX and X-ray diffraction analysis (XRD), as shown in Fig. 3c and d. There are no intermetallic compounds such as Cu_6_Sn_5_/Cu_3_Sn/Ag_3_Sn in the interfacial layer, indicating that no reactions of Cu, Sn, and Ag occurred during electroplating and PVD, which are beneficial for the preparation of dense three-dimensional network structure joints with IMC-reinforced Cu particles.

### Phase composition evolution and micromorphology of three-dimensional network structure of Cu@Sn@Ag TLPS joints

**Fig. 4:**
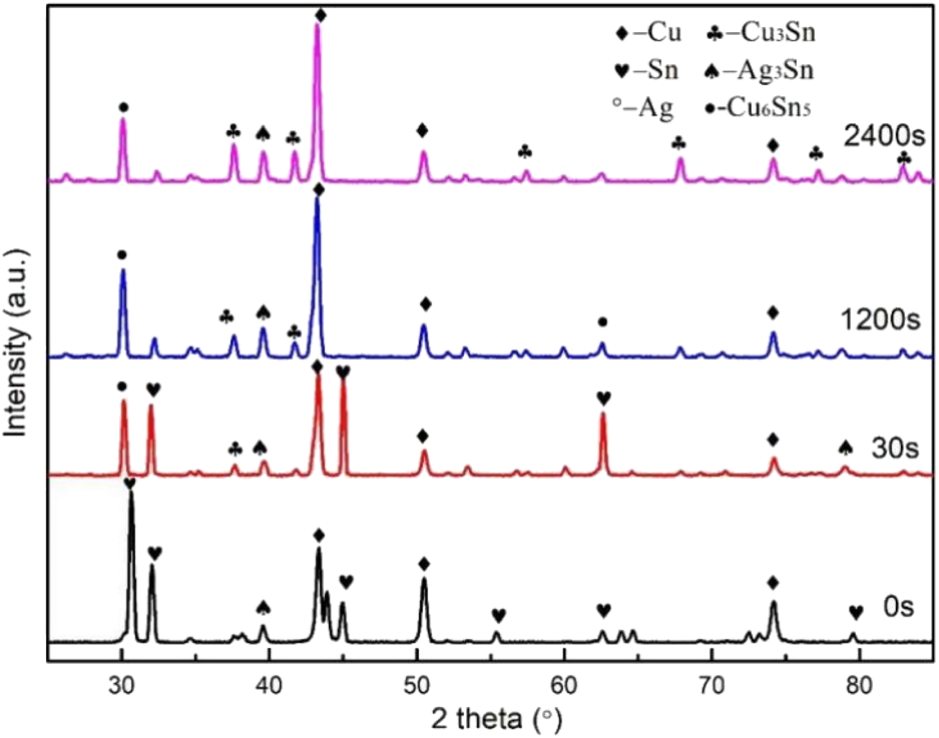
XRD results of the Cu@Sn@Ag preform TLPS for 0 s, 30 s, 1200 s, and 2400 s at 280 °C.

**Fig. 5:**
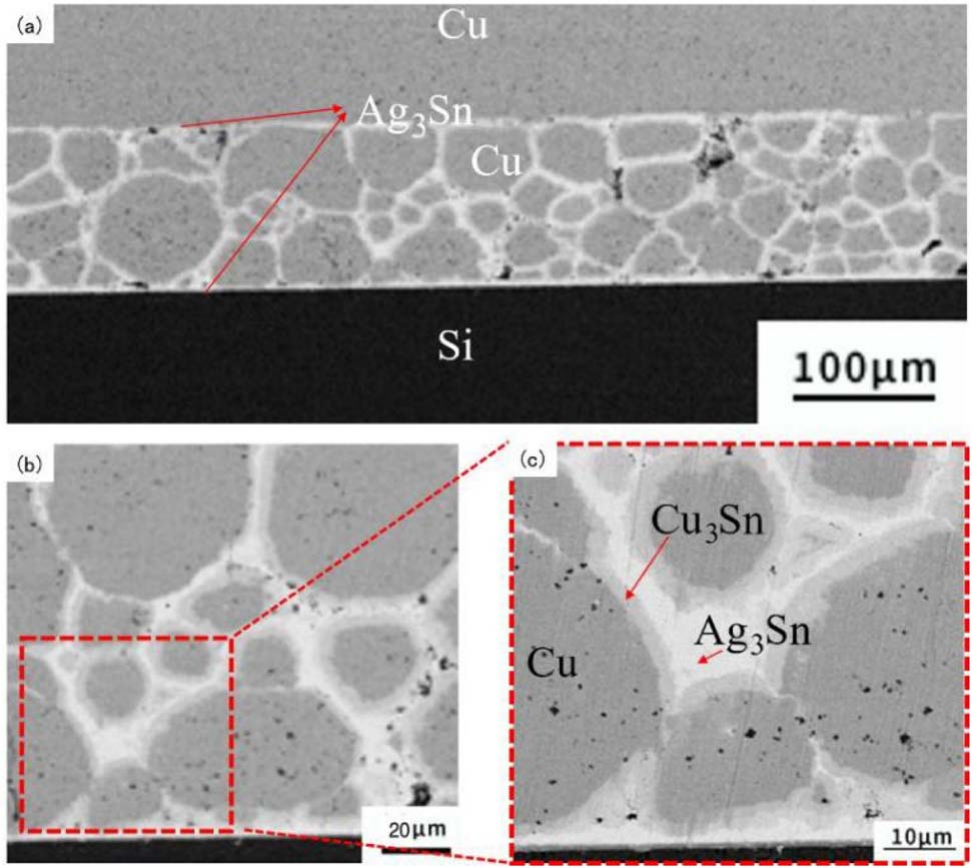
Cross-sectional morphology of Si chips bonded with Cu@Sn@Ag preform at 280 °C for 20 min: (a) overall morphology of the weld seam, (b) partial view of the joint structure, and (c) distribution of Cu/Cu_3_Sn/Ag_3_Sn phases in the joint.

The Cu@Sn@Ag composite powder was subjected to transient liquid-phase heat treatment at 280 °C for different holding times, and the phase evolution of the Cu@Sn@Ag preform at different holding times was characterized by XRD. As shown in Fig. 4, Cu, Ag, and Sn peaks were detected in the XRD patterns before the transient liquid-phase sintering process. After the preform was heated at 280 °C for 30 s, the Sn phase in the interface was completely consumed, with some generating Cu_6_Sn_5_ around Cu particles and the rest reacting with molten Sn to generate Ag_3_Sn grains. As the reaction time prolonged, the scalloped Ag_3_Sn grains continued to grow and underwent a solid phase diffusion process before densifying the interface microstructure. When the isothermal solidification time was 2400 s, the reaction was complete, and there was no Cu_6_Sn_5_ phase in the interface bulk phase except Ag_3_Sn, Cu_3_Sn, and Cu phases. Finally, the three-dimensional network structure Cu@Cu_3_Sn@Ag_3_Sn joint with high thermal stability was formed, as shown in Fig. 5. Further, the Cu particles were wrapped by a Cu_3_Sn layer with a thickness of 1–3 μm, and the outer network structure is a dense Ag_3_Sn phase with a thickness of 2–3 μm, and no observable holes were found in the local amplification region.

### Mechanical, thermal and electrical characteristics of the Cu@Sn@Ag TLPS joint

#### Shear strength of welding interface of Si device

**Fig. 6:**
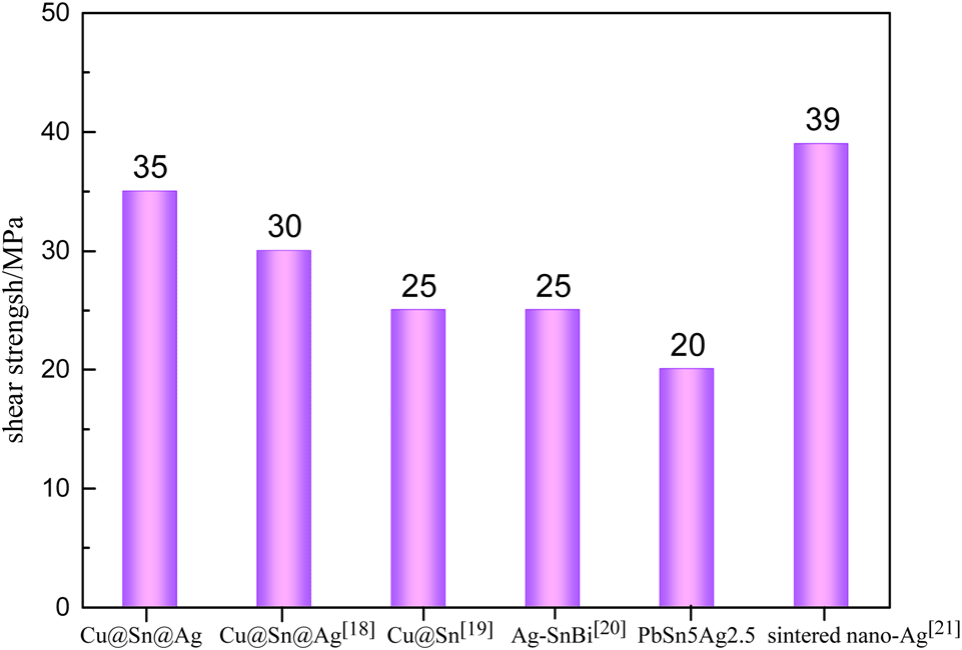
Comparison of shear strength of Cu@Sn@Ag preform and other solders.

Shear strength testing is one of the main methods to evaluate the reliability of chip welding. Herein, six samples of Cu@Sn@Ag solder and six samples of PbSn5Ag2.5 solder were subjected to shear strength testing, and the measured shear strength of the welding layer and its comparison with other solders are as displayed in Fig. 6. The average shear strength of the Cu@Sn@Ag solder joint is ∼35 MPa, which exceeds that of the same system soldered with, namely, Cu@Sn@Ag,^18^ Cu@Sn,^19^ Ag–SnBi,^20^ and commercial PbSn5Ag2.5 solder joints, and is similar to that of a sintered nanosilver^21^ solder joint.

The superior shear strength was attributed to the unique cladding structure of the Cu@Sn@Ag solder. This structure shortens the Cu/Sn/Ag element interdiffusive channel, accelerating the formation of Cu_3_Sn and Ag_3_Sn interfacial metallic compounds. This continuous transformation from Cu_6_Sn_5_ to Cu_3_Sn ultimately yields a dense three-dimensional network structure of Cu_3_Sn/Ag_3_Sn/Cu during the welding process with a short dwelling duration; this improves the strength and toughness of IMC joints. Moreover, the Sn layer electroplated on both sides of the preform catalyzes the reaction between the solder joint, DBC substrate, and copper block, improving the interface density and solving the problem of poor coplanarity. The strength of the welding layer can be increased by employing one material that produces compressive stress, whereas the other material produces equal tensile stress. Finally, the obtained welding layer of Cu@Sn@Ag can improve the reliability of device in high-temperature and high-power regions.

#### Thermal resistance of Si devices

**Fig. 7:**
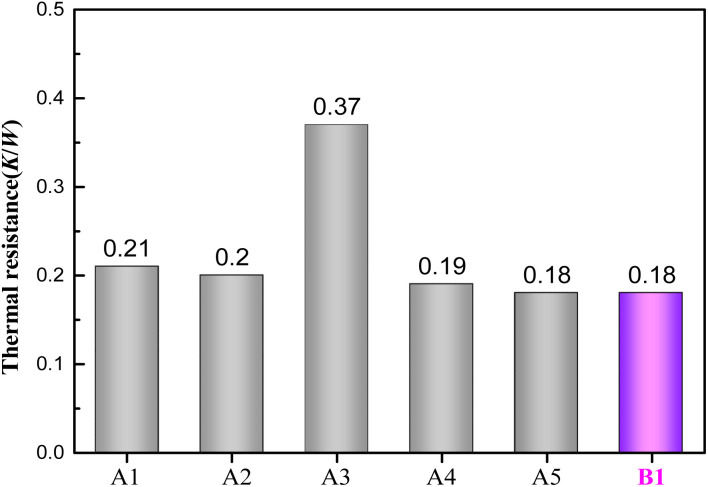
Thermal resistance of Si devices based on different solders (A1–A3: unoptimized Cu@Sn@Ag preform; A4, A5: optimized Cu@Sn@Ag preform; B1: PbSn5Ag2.5 preform).

The thermal resistance of devices based on different die attachment materials is depicted in Fig. 7. Si devices, A4 and A5, were assembled using optimized Cu@Sn@Ag preforms obtained after grinding the indenter and gasket and adjusting the compression time and pressure. In contrast, Si devices A1–A3 were assembled using unoptimized Cu@Sn@Ag preforms, and device B1 was assembled using a commercial PbSn5Ag2.5 preform. The thermal resistance of Si device B1 assembled using commercial PbSn5Ag2.5 solder is 0.18 K W^−1^. However, owing to the uniformity of the thickness of the Cu@Sn@Ag solder, the thermal resistance of devices assembled with this Cu@Sn@Ag solder is tremendously different, and the maximum thermal resistance is 0.37 K W^−1^. The porosity of the solder layers of devices A4 and A5 assembled using the optimized Cu@Sn@Ag preforms is ∼6–8%, and their maximum thermal resistance is 0.19 K W^−1^, which is similar to that of the commercial PbSn5Ag2.5 solder assembly devices. This is attributed to the uniformly distributed Cu particles inside the Cu@Sn@Ag solder layer, thus greatly improving the overall thermal conductivity of the solder (144 W m^−1^ K^−1^),^22^ which is nearly five times that of the commercial PbSn5Ag2.5 solder (26 W m^−1^ K^−1^).^23^ Owing to the uneven thickness, the Cu@Sn@Ag layer exhibits thermal resistance that exceeds that of the commercial PbSn5Ag2.5 solder layer. However, this defect is compensated by the excellent thermal conductivity of Cu@Sn@Ag. The thermal resistance of device A (30.37 K W^−1^) is relatively high owing to the high porosity of the solder layer, which affects the heat conduction of the chip.

#### Power cycle reliability of Si devices

For the diode, a higher forward voltage drop implies a greater dissipated power, and the increase of the dissipated power entails the increase of the junction temperature of the device. For every 10 °C increase in the junction temperature, the failure efficiency would be doubled,^24^ and the increase of the junction temperature will entail the decline of chip performance and finally cause the failure of the device. Therefore, the device with lower forward voltage drop will have higher thermal reliability.

**Fig. 8:**
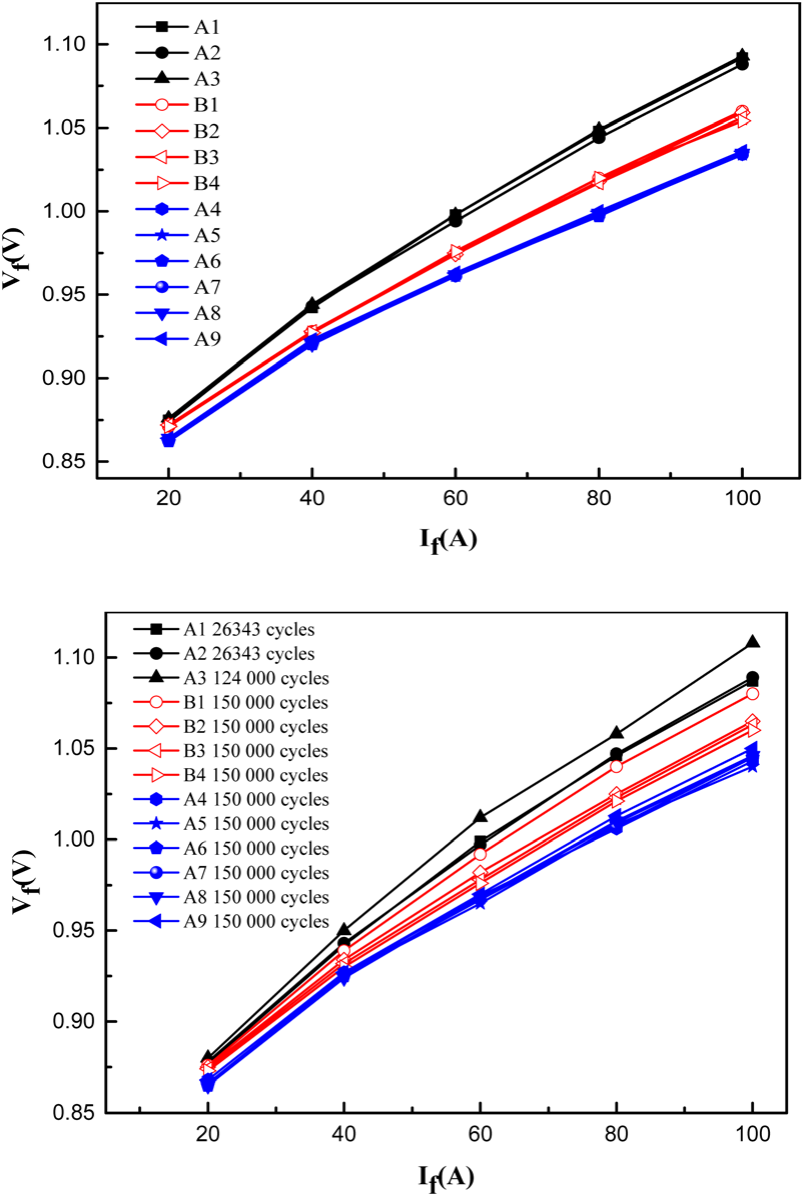
*I*–*V* curves of Si devices before and after power cycling (A1–A3: unoptimized Cu@Sn@Ag preform, B1–B4: PbSn5Ag2.5 preform; A4–A9: optimized Cu@Sn@Ag preform).

Fig. 8a and b show the *I*–*V* curve of Si devices with different preforms before and after power cycling, respectively. The overlap of the measured *I*–*V* curves of devices A4–A9 with the optimized preforms is comparable to that of the devices with the commercial PbSn5Ag2.5 preforms, even with a miniscule change trend and lower forward voltage drop, which proves that the Si devices welded with optimized Cu@Sn@Ag preforms have appreciable quality uniformity.

The input current of 100 A is provided by an external DC power supply. During the power cycle test, the electrical performance and temperature parameters of the device will change, and a 5% increase in forward voltage drop after power cycle is generally employed as the criterion for device failure.^25^ The parameters of the power cycle test have been set as follows: constant on-time 1 s and off-time 1 s. When the chip temperature reaches the set maximum temperature of 125 °C, the Si device is turned off, and the chip temperature drops to the set minimum temperature of 65 °C, with the assistance of the radiator to complete the cycle process. The continuous cycle temperature difference (60 °C) will affect thermal stress and fatigue, and ultimately the reliability of the device in actual work will be calculated.

**Fig. 9:**
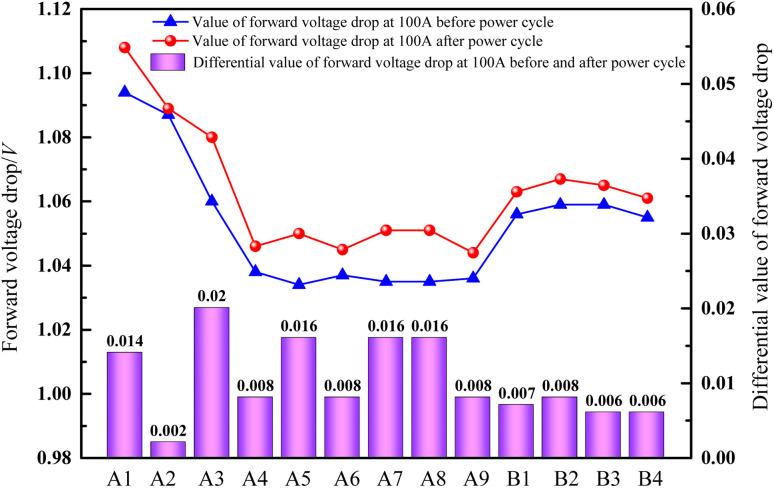
Change in forward voltage drop of Si devices at 100 A before and after power cycling (A1–A3 unoptimized: Cu@Sn@Ag preform; A4–A9: optimized Cu@Sn@Ag preform; B1–B4: PbSn5Ag2.5 preform).

Further analysis of the *I*–*V* curves, before and after the power cycle, displays that there is little difference in the forward voltage drop of Si devices assembled with different solder pads. The growth rate of the forward voltage drop is below 2% (Fig. 9), before and after the power cycle at 100 A. The stability of the swing amplitude of the forward voltage drop difference of the Si device, which is welded using Cu@Sn@Ag, generally falls below that of the Si device welded using commercial PbSn5Ag2.5. The forward voltage drops of devices A4–A9, before and after the power cycle, falls below those of the four commercial PbSn5Ag2.5 solders, which shows that the optimized Cu@Sn@Ag preforms can meet the reliability requirements for the long-term operation of electronic devices with better application prospects.

## Conclusions

A novel type of Cu@Sn@Ag preform is developed, and a commercial Si chip is welded onto a DBC board using the developed preform and a commercial PbSn5Ag2.5 preform. The effects of different die attachment materials on the thermal, mechanical, and electrical properties and reliability of the Si device have been studied. It is found that the quality of preparation of the Cu@Sn@Ag preform has a tremendous influence on the shear strength and power cycle ability of the whole device.

(1) Cu@Sn@Ag dual-core composite powder and its TLPS joint were prepared. The phase composition and micromorphology evolution of the three-dimensional network structure of Cu@Cu_3_Sn@Ag_3_Sn dense joint were investigated, which were used to obtain the optimized soldering parameters.

(2) The unique cladding structure of the Cu@Sn@Ag solder makes it possible to obtain a dense three-dimensional network structure of Cu_3_Sn/Ag_3_Sn/Cu with excellent mechanical and thermoelectric properties. The shear strength of the welding layer of Cu@Sn@Ag welded with double-sided Sn electroplating can reach 35 MPa, which exceeds that of PbSn5Ag2.5 solder and other welding layers in the same system. The minimum thermal resistance of the corresponding device is ∼0.18 K W^−1^, close to that of the PbSn5Ag2.5 joint.

(3) The *I*–*V* curves of the Si devices assembled by employing the optimized Cu@Sn@Ag and PbSn5Ag2.5 solders are approximately the same before and after 150 000 power cycles. The forward voltage drop of the Si device welded with the optimized Cu@Sn@Ag solder is below that of the PbSn5Ag2.5 solder, which demonstrates its higher reliability during use.

## Author contributions

Conceptualization, Hongyan Xu; methodology, Hongyan Xu, and Honghui Zhang; resources, Hongyan Xu, and Honghui Zhang; writing—original draft preparation, Honghui Zhang, Tianwen Wang; writing—review and editing, Hongyan Xu, Honghui Zhang, Tianwen Wang and Sheng Wang; supervision, Hongyan Xu, Tianwen Wang and Sheng Wang; project administration, Hongyan Xu; funding acquisition, Hongyan Xu. All authors have read and agreed to the published version of the manuscript.

## Conflicts of interest

There are no conflicts to declare.

## Supplementary Material
